# Sequence analysis of origins of replication in the *Saccharomyces cerevisiae* genomes

**DOI:** 10.3389/fmicb.2014.00574

**Published:** 2014-11-18

**Authors:** Wen-Chao Li, Zhe-Jin Zhong, Pan-Pan Zhu, En-Ze Deng, Hui Ding, Wei Chen, Hao Lin

**Affiliations:** ^1^Key Laboratory for Neuro-Information of Ministry of Education, Center of Bioinformatics, School of Life Science and Technology, University of Electronic Science and Technology of ChinaChengdu, China; ^2^Department of Physics, School of Sciences and Center for Genomics and Computational Biology, Hebei United UniversityTangshan, China

**Keywords:** *Saccharomyces cerevisiae*, origin of replication, GC profile, GC skew, information redundancy, distribution of ORIs

## Abstract

DNA replication is a highly precise process that is initiated from origins of replication (ORIs) and is regulated by a set of regulatory proteins. The mining of DNA sequence information will be not only beneficial for understanding the regulatory mechanism of replication initiation but also for accurately identifying ORIs. In this study, the GC profile and GC skew were calculated to analyze the compositional bias in the *Saccharomyces cerevisiae* genome. We found that the GC profile in the region of ORIs is significantly lower than that in the flanking regions. By calculating the information redundancy, an estimation of the correlation of nucleotides, we found that the intensity of adjoining correlation in ORIs is dramatically higher than that in flanking regions. Furthermore, the relationships between ORIs and nucleosomes as well as transcription start sites were investigated. Results showed that ORIs are usually not occupied by nucleosomes. Finally, we calculated the distribution of ORIs in yeast chromosomes and found that most ORIs are in transcription terminal regions. We hope that these results will contribute to the identification of ORIs and the study of DNA replication mechanisms.

## INTRODUCTION

The well-known replication theory was proposed in 1963 based on a large number of experiments using the sexual system of *Escherichia coli* (Jacob et al., 1963). DNA replication is an orchestrated process. When a cell enters the S phase of replication, the DNA double helix of this cell is unwound. Then, replication forks are generated to allow the DNA synthesis machinery to copy each DNA strand in a bidirectional manner. In the process of replication, the specific regions that are responsible for the initiation of the replication of the genome are called origin of replication (ORI) regions. These regions are recognized by the origin recognition complex (ORC). The DNA replication process is usually activated only once per cell cycle to avoid amplification and maintain genome integrity (Cayrou et al., 2012).

Although most of bacterial genomes have only a single ORI region (Gao and Zhang, 2007) and some archaea use more than one ORI region to initiate DNA replication (Luo et al., 2014), the fungus, *Saccharomyces cerevisiae* (*S. cerevisiae*) has multiple ORIs on its chromosomes to perform complete replication in a reasonable period of time because of the large size of its genomes and the limitation of nucleotide incorporation during DNA synthesis. Therefore, predicting ORIs is more difficult in the *S. cerevisiae* genome than that in bacterial genomes. Several experiments have revealed that the activity of ORIs in yeast depends on a *cis*-acting replicator sequence termed autonomous replication sequence (ARS). These regulatory sequences are generally found in AT-rich regions in yeast genome. The ARS generally contains three domains: A, B, and C. An essential ARS consensus sequence (ACS) (T/A)TTTAT(A/G)TTT(T/A) usually appears in the A domain (Wu et al., 2014). The B domain contains a number of short sequence motifs that contribute to origin activity (Dhar et al., 2012). The motifs in the C domain are responsible for the interaction between DNA and regulatory proteins (Crampton et al., 2008). However, these motif sequences are not conserved enough to be used to identify ORIs (Nieduszynski et al., 2006). Thus, the discovery of the hidden intrinsic characteristics at the sequence level is helpful not only for understanding the regulatory mechanism but also for accurately identifying ORIs.

With the accumulation of experimental data (Levitsky et al., 2005; Yamashita et al., 2011; Gao et al., 2012), some researchers have analyzed features of replication. Recently, by analyzing four highly active origins, Chang et al. (2011) revealed that sequences adjacent to the ACS contributed substantially to origin activity and ORC binding. Yin et al. (2009) found that the nucleosome depletion regions are preferentially permissive for replication and proposed that the ORI organization imposed by nucleosome positioning is phylogenetically widespread in eukaryotes. DNA structure may also influence the distribution of ORIs. Chen et al. (2012) found that the DNA bendability and cleavage intensity in ORIs are dramatically lower than those in both upstream and downstream regions of ORIs.

Although some characteristics of ORIs have been described, the available information about ORIs is still far from satisfactory. Therefore, to clarify replication mechanisms, it is still necessary to discover the intrinsic characteristics of ORIs. With this in mind, we performed a series of analyses to investigate the composition bias and correlation of nucleotides in ORIs, the distribution of ORIs in genomes, and the relationships between ORIs and regulatory elements.

## MATERIALS AND METHODS

### DATASETS

The *S. cerevisiae* ORIs were collected from OriDB (Siow et al., 2012; http://www.oridb.org/). The confidence of the ORI data has three levels: confirmed, likely, and dubious. To provide a reliable and high-quality dataset, only the 410 experimentally confirmed ORIs were selected and used in the following analysis.

The complete *S. cerevisiae* genome was downloaded from GenBank (Benson et al., 2013). The 5015 transcription start sites (TSSs) of *S. cerevisiae* were previously published (Lee et al., 2007). The *in vitro* nucleosome data and nucleosome data from three growth conditions [ethanol, yeast extract, peptone, and dextrose (YPD) medium, and galactose] were previously reported (Yuan et al., 2005; Lee et al., 2007; Kaplan et al., 2009)

### SEQUENCE COMPOSITION ANALYSIS

The GC profile represents the variation in GC content along the genomic sequence (Gao and Zhang, 2006), which can be defined by the following equation (Zhang et al., 2005; Xing et al., 2014):

(1) $$GC\,\;\,profile\left[i\right]=\frac{f_{i}(G)+f_{i}\left(C\right)}{f_{i}(A)+f_{i}(C)+f_{i}(G)+f_{i}\left(T\right)}$$

where *f_i_*(A), *f_i_*(C), *f_i_*(G), and *f_i_*(T) are the frequencies of adenine(A), cytosine(C), guanine (G), and thymine(T), respectively, in the *i*-th sliding window along the sequence. The range of values for the GC profile is between 0 and +1. Values ranging from 0 to 0.5 indicate that the GC content in the *i*-th sliding window is lower than the AT content, while values ranging from 0.5 to 1 indicate that the GC content in the *i*-th sliding window is higher than the AT content.

GC skew was the first proposed computational method to identify ORIs in bacterial genomes (Lobry, 1996a,b). For a given sequence, the GC skew is defined by the following equation (McLean et al., 1998):

(2) $$GC\,\;skew\left[i\right]=\frac{f_{i}(G)-f_{i}\left(C\right)}{f_{i}(G)+f_{i}\left(C\right)}$$

where *f_i_*(C) and *f_i_*(G) are the frequencies of cytosine(C), and guanine (G) in the *i*-th sliding window along a sequence, respectively. The range of values for GC skew is between -1 and +1. Values ranging from -1 to 0 indicate that *f_i_*(G) < *f_i_*(C), and values ranging from 0 to +1 indicate that *f_i_*(G) > *f_i_*(C).

### INFORMATION REDUNDANCY

As a genetic language, the nucleic acid sequence can be investigated through an information-theoretic method (Luo et al., 1998). In recent years, informational entropy was widely applied in the recognition and evolution research of DNA sequences (Grosse et al., 2000; Yu and Jiang, 2001; Otu and Sayood, 2003; Xing et al., 2013). The average mutual information profile is an excellent candidate for a species signature (Bauer et al., 2008). Based on these studies, we introduced the *k*-order information redundancy, which can be defined as follows (Luo et al., 1998):

(3) $$D_{k+2}=2H+\underset{i,j}{\Sigma }p_{i(k)j}\,\;\log_{2}\,\;p_{i(k)j\,}\,\;\;\;\;\;\;\;\;\;\;\;\;\;\;\;\;\;\;\;\;\;\;\;\;\;\;\;\;\;k=0,1,2,...$$

where *p_i_*_(_*_k_*_)_*_j_* is the joint probability of base *j* occurring after base *i* at a distance *k* along the sequence. The term *k*= 0 indicates the adjacent correlation between two bases. *D_k_*_+2_ describes the divergence of the sequence from independence and the correlation between nucleotides with the gap of *k* nucleotides. In general, the larger the *D_k_*_+2_ value is, the stronger the divergence degree of the sequence from independence is. The *H* value is the informational entropy and is defined by the following equation

(4) $$H=-\underset{a}{\Sigma }p_{a}\,\;\log_{2}\,\;p_{a}$$

where *p_a_* is the probability of base *a* (*a* = A, G, C, or T) occurring in the sequence.

## RESULTS AND DISCUSSION

### GC CONTENT SURROUNDING ORIs

DNA sequence information is the most basic but important genetic information. It also plays an important role in the determination of ORIs in the *S. cerevisiae* genome. However, the extent to which ORIs are determined *in vivo* by *cis*-acting sequence is poorly understood. To investigate the compositional bias of ORIs, we calculated the GC content of 300 bp of each ORI. As a comparison, the GC content of the genome sequence was also calculated by using a window of 300 bp with a step of 300 bp. The mean GC content of ORIs is 0.3168 (SD = 0.23 × 10^-2^), which is significantly lower (*P* < 2.3 × e^-133^, Mann–Whitney *U*-test) than the genome-wide GC content (0.3796; SD = 0.24 × 10^-2^). In other words, ORIs are AT-rich. The high AT content of ORI sequences contributes to the opening of the DNA double helix structure for the initiation of DNA replication.

### GC PROFILE AND GC-SKEW SURROUNDING ORI

**FIGURE 1:**
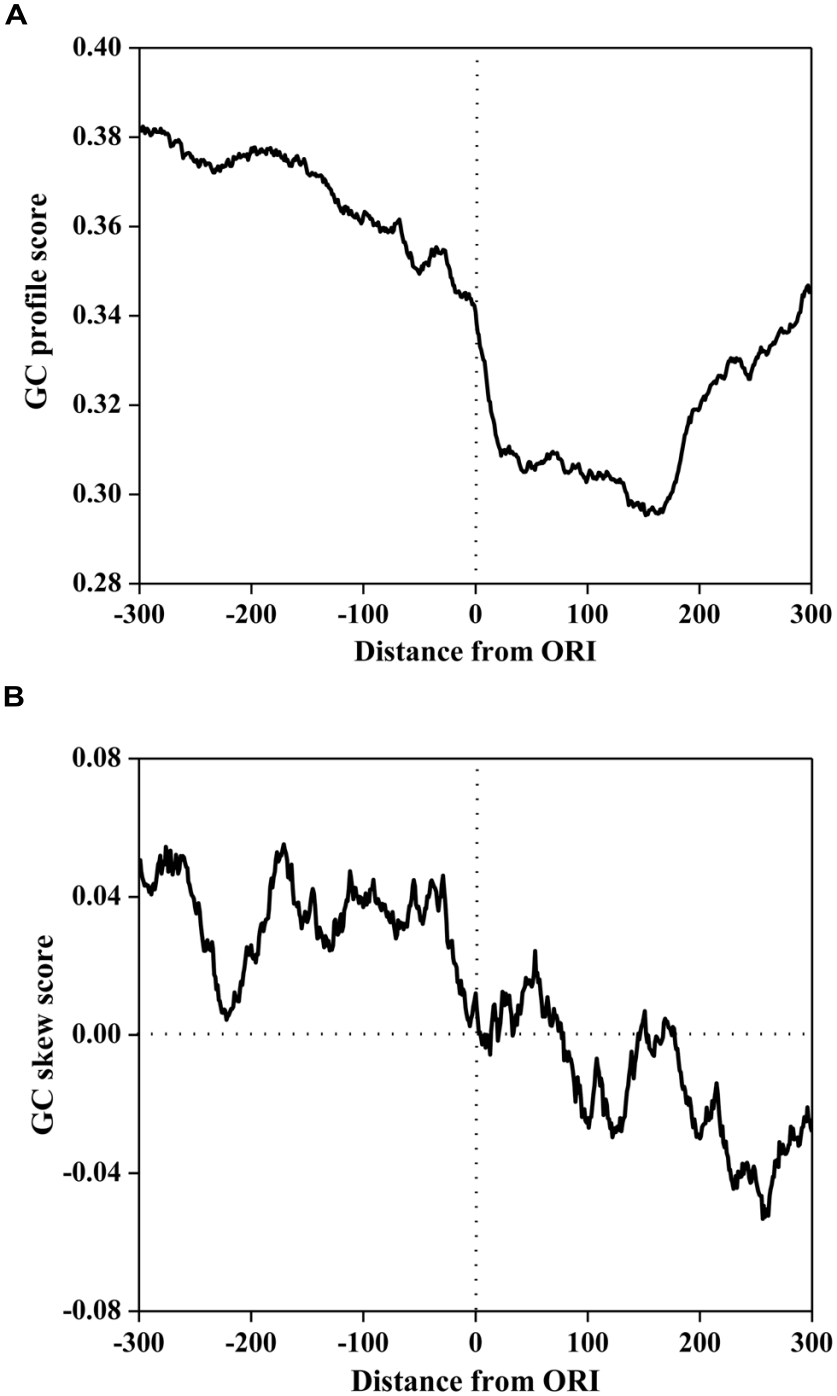
**The GC profile (A) and the GC skew (**B**) of origins of replication (in ORIs) and surrounding regions.** The profiles are plotted using a 50-bp sliding window with a 1-bp step. The horizontal axis represents the nucleotide position, which ranges from -300 to +300 bp relative to ORIs (denoted as 0). The vertical axis represents the GC content score **(A)** and GC skew score **(B)**.

To investigate the compositional bias, the GC profile and GC skew surrounding ORIs was calculated using a 50 bp sliding window with a step of 1 bp. The average scores of the GC profile and GC-skew are plotted in **Figure 1**. As illustrated in **Figure 1A**, the score of the GC profile in the ORI regions was statistically lower than that in the surrounding regions (*P* < 2.0 × e^-86^, Mann-Whitney *U*-test).

To further investigate the sequence mode of ORI sequences, MEME (Multiple Em for Motif Elicitation; Bailey and Elkan, 1994) was used to discover the consensus motifs in ORI sequences. We found that the consensus sequences are all AT-rich motifs. It has been reported that ORIs contain some AT-rich elements for interactions with regulatory proteins (Reeves and Beckerbauer, 2001; Takahashi et al., 2003). Previous research demonstrated that the information encoded in the high AT content can be recognized by the Orc4 subunit of ORC (Mojardin et al., 2013). This can be attributed to the enrichment of the ACS around ORIs in *S. cerevisiae*, which is an AT-rich motif that contains the binding site for ORC. Recent research also revealed that a conspicuous feature of a replication regulatory protein was the presence of nine AT-hook domains in its amino terminus (Chuang and Kelly, 1999) that were essential for the binding of ORC to ORIs.

However, the GC skew in **Figure 1B** displays a different trend. The GC skew score in the core ORI regions was statistically lower than that in the upstream regions (*P* < 2.3 × e^-80^, Mann-Whitney *U*-test), but higher than that in the downstream regions (*P* < 5.0 × e^-40^, Mann-Whitney *U*-test). We noticed that the GC skew score conversed from positive to negative at the 0^th^ site corresponding to the DNA replication initiation site. In bacterial genomes, GC skew changes sign at the boundaries of the two replichores, which correspond to the DNA replication origin or terminus (Lobry, 1996a; Necsulea and Lobry, 2007). Thus, our finding implies that the *S. cerevisiae* genome may have a replication mechanism that is similar to that of bacterial genomes.

### CORRELATION OF NUCLEOTIDES SURROUNDING ORIs

**FIGURE 2:**
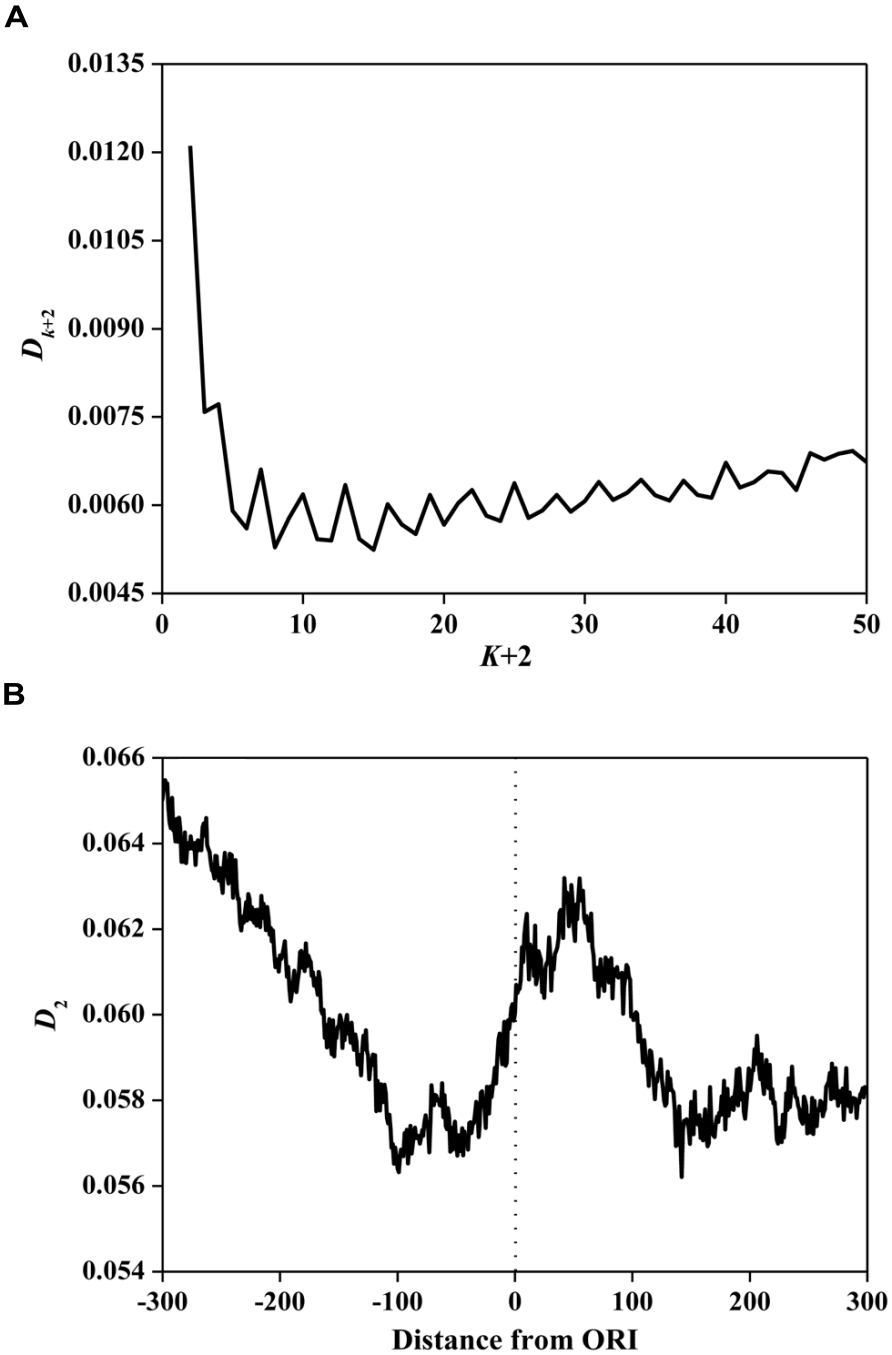
**(A)** Average *D_k_*_+2_ vs. *k*+2 for the ORI sequences. The horizontal axis represents the gap of *k*+2. The vertical axis represents the value of *D_k_*_+2_. **(B)** The distribution of *D*_2_ surrounding ORIs. The horizontal axis represents the nucleotide position, which ranges from -300 bp to +300 bp relative to ORIs (denoted as 0). The vertical axis represents the value of *D*_2_.

Based on Eq. 3, we calculated information redundancies *D_k+_*_2_ of ORI sequences. The average values are illustrated in **Figure 2A**. The main maxima for most ORI sequences are located on *D*_2_. This result demonstrates that *D*_2_ is the maximum among all considered *D_k_*_+2_ (*k*= 0, 1, …, 48), indicating that ORI sequences have a short-range dominance of base correlations. Subsequently, we calculated *D*_2_ in a 150 bp window with a step of 1 bp for ORI sequences. As shown in **Figure 2B**, a peak near the ORIs and two valleys flanking the ORIs were observed, suggesting that the ORI sequences have very strong short-range correlations. It has been reported that *D*_2_ is correlated with the evolutionary active region (Du et al., 2006). As a special region in the replication process, ORIs have a high probability of deletion, insertion, and mismatch (Umar and Kunkel, 1996). Thus, the evolutionary force reflected by the *D*_2_ constraint indicates the diversity of ORI sequences. However, the evolutionary mechanism of fungi ORIs needs further investigation.

### DISTRIBUTION OF ORIs IN THE GENOME

It is widely accepted that functional regions are not randomly distributed in the genome (Zhang et al., 2007). Based on this hypothesis, we statistically analyzed the distribution of ORIs in the yeast genome.

**FIGURE 3:**
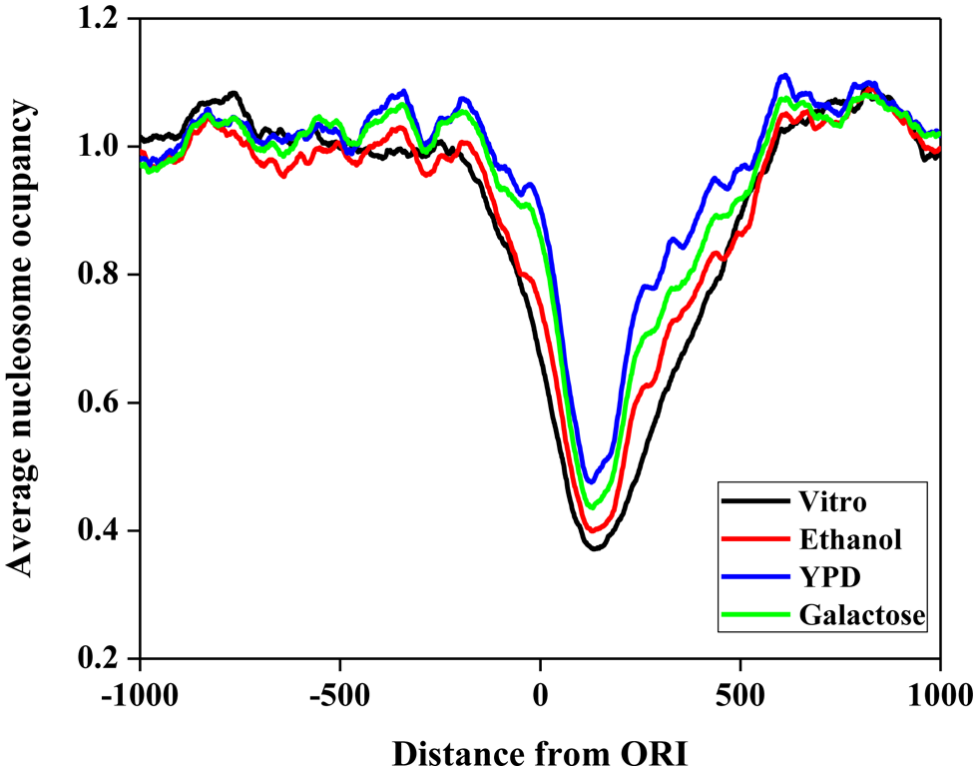
**Nucleosome occupancy around ORIs.** The black curve represents the *in vitro* data. The red, blue, and green curves represent *in vivo* experimental maps for three growth conditions (ethanol, yeast extract, peptone, and dextrose medium [YPD] and galactose).

First, we investigated the position relationship between ORIs and nucleosomes. Nucleosomes are the elementary units of chromatin organization and are composed of a ∼147 bp stretch of DNA that is tightly wrapped around a histone core (Richmond and Davey, 2003; Segal et al., 2006). Nucleosome positioning affects nearly every cellular process that requires protein access to genomic DNA (Lee et al., 2007; Kaplan et al., 2009). Thus, it is worth studying the nucleosome occupancy around ORIs. To examine the distribution of nucleosomes around ORIs, we selected regions from -1000 to 1000 bp flanking ORIs and then mapped the nucleosomes in these regions. The average nucleosome occupancy scores surrounding ORIs *in vitro* and *in vivo* (ethanol, YPD, and galactose) are shown in **Figure 3**. The nucleosome occupancies around ORIs both *in vitro* and *in vivo* display a similar tendency: i.e., the nucleosome occupancy scores in ORIs are significantly lower than those in flanking regions, indicating that ORIs always appear in the nucleosome-free regions. This result can be explained as follows: once wrapped around the histone core, it is difficult for regulatory proteins to access the regions, which makes it difficult to open the DNA double helix (Kass and Wolffe, 1998).

Gene transcription also requires the opening of the DNA double helix. Thus, there are coupling effects between ORIs and promoters. In fact, several studies focused on replication–transcription interactions (Rocha, 2004; Sequeira-Mendes and Gomez, 2012; Helmrich et al., 2013; Lubelsky et al., 2014). Here, the distance between ORIs and TSSs in the yeast genome was calculated. For over 31.46% of cases, the distance between ORI and TSS was less than 500 bp. These promoters are also AT-rich sequences (Lee et al., 2001). Thus, these promoters might share elements with ORIs.

Origins of replications are associated with bias in gene density (Necsulea et al., 2009). To further investigate the relationship between replication and transcription, we analyzed the distribution of ORIs in three kinds of intergenic regions. We obtained 2770 tandem, 1514 divergent, and 1497 convergent intergenic regions based on the orientations of the adjacent gene pair from the GenBank database. The tandem and divergent intergenic regions usually contain promoters; especially, each divergent intergenic region has two promoters for the transcription of two genes, whereas no promoter appears in convergent intergenic regions. By mapping ORIs in these regions, we found that 12.9% of ORIs are located in convergent regions, 25.1% are located in tandem regions, and 12.9% are located in divergent regions. The remaining ORIs (about 46.8%) overlap with coding regions, including 16.3% that are found in the tail of coding regions and 6.6% that are in the head of genes. These results suggest that most ORIs are not biased to transcription start regions, which may guarantee the coordination of replication and transcription.

### PREDICTION OF ORIs

The aim of the above statistical analysis was to gain intrinsic observations to understand the replication initiation mechanism and to provide enough information for ORI prediction. Thus, we evaluated the predicted accuracies of the GC profile, GC skew, information redundancy *D*_2_, and nucleosome occupancy to discriminate the ORIs from non-ORIs using a support vector machine. Here, 300 bp of each ORI was selected as the positive set, while the 300 bp upstream of ORIs was extracted as the negative set. The 10-fold cross-validated results are recorded in **Table 1**. It is obvious that the nucleosome occupancy feature can more accurately predict ORIs than GC skew and *D*_2_. The comparative accuracy was also obtained with the GC profile. However, these results are still far from satisfactory. The features of GC profile, GC skew, and *D_2_* are based on the nucleotide sequence content, in which little sequence-order effect was considered. In the future, we will consider the sequence-order effect to improve the prediction quality.

**Table 1:** Predicted results of different parameters using a support vector machine^**a**^.

Method	Performance evaluation^b^		
	*Sn*	*Sp*	*Acc*
GC profile	0.7605	0.7728	0.7667
GC skew	0.6247	0.5778	0.6012
*D*_ 2_	0.5309	0.5704	0.5506
Nucleosome (*in vitro*)	0.7448	0.7575	0.7511
Nucleosome (ethanol)	0.7071	0.7840	0.7456
Nucleosome (YPD)	0.7567	0.7811	0.7689
Nucleosome (galactose)	0.7485	0.7910	0.7697

*^a^The software package LIBSVM (version 3.17) was used to implement the support vector machine. The best separating hyperplane was constructed using the basis of radial basis kernel function. The regularization parameter *C* and the kernel width parameter γ were optimized using the grid-search approach.*

*^b^The three metrics, sensitivity (*Sn*), specificity (*Sp*), and overall accuracy (*Acc*), we re defined as Sn = TP / (TP+FN), Sp = TN / (TN+FP), and Acc = (TP+TN) / (TP+TN+FP+FN), respectively, where *TP* denotes the number of correctly predicted ORIs, *FN* denotes the number of ORIs that were predicted as non-ORIs, *FP* denotes the number of non-ORIs that were predicted as ORIs, and *TN* denotes the number of correctly predicted non-ORIs.*

## CONCLUSION

Despite several studies focusing on DNA replication, the mechanism of replication initiation remains elusive. This study focused on the ORIs of *S. cerevisiae* and systematically analyzed the sequences surrounding ORIs. We found that the sequence around ORIs had a lower GC profile score and a higher nucleotide correlation than the sequence in flanking regions. DNA replication is a highly regulated process that relies on interactions between regulatory proteins and DNA sequences. The AT-rich motif is easily recognized by ORC. By studying the distribution of ORIs in genomes, we found that DNA replication initiation usually occurs in nucleosome-free regions. The short distance between ORIs and TSSs suggested that the expression of genes may be influenced by DNA replication. We expect that the observed properties of ORIs in this work will influence research related to ORIs and provide novel insights into regulatory mechanisms of DNA replication.

## Conflict of Interest Statement

The authors declare that the research was conducted in the absence of any commercial or financial relationships that could be construed as a potential conflict of interest.
